# Biocomposites of Alginate, Calcium Polyphosphate, and Silver Nanostructures: Antibacterial Systems for Bone Regeneration Applications

**DOI:** 10.3390/ph19060917

**Published:** 2026-06-10

**Authors:** Joalen Pereira do Monte, Rafael B. G. Pessoa, Adriana Fontes, Beate S. Santos, Giovannia A. L. Pereira, Goreti Pereira

**Affiliations:** 1 Department of Fundamental Chemistry, Federal University of Pernambuco, Recife 50740-560, Brazil; joalen.monte@ufpe.br; 2 Department of Biochemistry, Federal University of Pernambuco, Recife 50670-901, Brazil; 3 Department of Biophysics and Radiobiology, Federal University of Pernambuco, Recife 50670-901, Brazil; adriana.fontes@ufpe.br; 4 Department of Pharmaceutical Sciences, Federal University of Pernambuco, Recife 50740-520, Brazil; beate.santos@ufpe.br; 5 Department of Chemistry & Centre for Environmental and Marine Studies (CESAM), University of Aveiro, 3810-193 Aveiro, Portugal

**Keywords:** hydroxyapatite, biomaterials, antimicrobial activity, antimicrobial resistance

## Abstract

**Background/Objectives**: Bone infection remains a severe clinical challenge characterized by recurrence, antimicrobial resistance, and high morbidity, driving the search for new therapeutic strategies. Despite advances in developing biomaterials with suitable biocompatibility, biodegradability, and structural properties, the lack of effective antibacterial activity continues to significantly limit the treatment of bone defects. To overcome this issue, we investigated the incorporation of silver-based nanostructures into calcium polyphosphate/alginate (CPP/Alg) matrices as an antibacterial reinforcement strategy for bone-related applications. **Methods**: Silver nanoparticles (AgNPs) were synthesized in aqueous medium via NaBH_4_-mediated chemical reduction, using either alginate (Alg) or sodium polyphosphate (PP) as stabilizing agents, enabling a comparative evaluation of biocompatible polymer- and polyphosphate-stabilized systems. Subsequently, AgNPs were incorporated into calcium polyphosphate/alginate (CPP/Alg) matrices to obtain Ag-containing composites. **Results**: The AgNPs exhibited spherical morphology, Zeta potential values ranging from −38.7 ± 0.2 to −23 ± 0.3 mV, and hydrodynamic diameters between 25.2 ± 0.2 and 143 ± 5 nm. Structural characterization of the biocomposites by X-ray diffraction confirmed hydroxyapatite as the major crystalline phase, while Raman spectroscopy revealed vibrational bands corresponding to both the inorganic and polymeric components. SEM revealed a dense, rough surface, and ICP-OES analysis confirmed the presence of Ag. Antibacterial activity assays demonstrated effective growth inhibition of *Staphylococcus aureus* and *Staphylococcus epidermidis*, with inhibition halos growing with increasing composite dosage. Notably, antibacterial activity was achieved at relatively low Ag contents, underscoring the efficiency of these biocomposites. **Conclusions**: These findings confirm the effective incorporation of AgNPs into the CPP/Alg matrix and support the classification of composites as promising antibacterial biomaterials for bone regeneration applications.

## 1. Introduction

The development of materials with suitable physicochemical and biological properties for clinical applications is a common focus of research in regenerative medicine and tissue engineering [1,2]. To address orthopedic, maxillofacial, and dental problems, several strategies have been adopted for treating bone defects, including autografts, allografts, moldable bone cements, and distraction osteogenesis. However, these approaches present limitations such as high donor-site morbidity, secondary damage, chronic pain, immune rejection, and an increased infection risk [3].

Postoperative infections are among the most common complications associated with bone graft use. The pathogens typically associated with bone infections belong to the Gram-positive cocci, such as staphylococci, enterococci, and streptococci, particularly *Staphylococcus aureus* and coagulase-negative staphylococci. Other microorganisms are also found in infectious processes, including Gram-negative bacilli such as *Escherichia coli*, *Proteus mirabilis*, and *Pseudomonas aeruginosa* [4,5]. Given the widespread use of antibiotics and the growing prevalence of antibiotic-resistant bacteria, alternative strategies to reduce infection risk are increasingly being explored. In this way, metallic nanostructures have attracted significant attention as antibacterial agents that enhance the functional performance of biomaterials without relying solely on systemic antibiotic therapy [6].

Although organic nanoparticles are useful as delivery carriers and often provide high biocompatibility and degradability, inorganic/metallic nanoparticles are particularly advantageous in bone-regeneration platforms. These inorganic nanostructures combine structural stability, tunable surface chemistry, great cell adhesion, and, in some cases, intrinsic antimicrobial activity. These features are especially relevant for infection-prone bone defects where local antibacterial functionality is crucial [6,7].

Among the different types of metallic nanoparticles explored in tissue engineering—such as aluminum (Al), gold (Au), iron (Fe), nickel (Ni), silver (Ag), copper (Cu), and zinc (Zn)—silver nanoparticles (AgNPs) stand out due to their remarkable antibacterial properties and well-documented efficacy against clinically relevant pathogens [6,8,9]. Rather than replacing antibiotics, AgNPs have been widely investigated as adjunct agents to improve the antimicrobial performance of biological matrices and synthetic scaffolds.

Recent studies have incorporated AgNPs into inorganic biomaterials or calcium phosphate-based composites, demonstrating enhanced antibacterial performance while maintaining cytocompatibility [7]. Calcium phosphate derivatives such as hydroxyapatite (HAp), tricalcium phosphate (TCP), dicalcium phosphate dihydrate (DCPD), and anhydrous dicalcium phosphate (DCPA), among others, are particularly attractive for bone regeneration due to their osteoconductive, osteoinductive, and biocompatible nature [10].

For example, Liu et al. investigated the antimicrobial activity and cytocompatibility of bone composites based on α-TCP- and DCPD incorporating AgNPs. Their results demonstrated that *S. aureus* was inhibited by 98% using AgNPs compared with the AgNP-free control. In addition to high antibacterial activity, the authors also assessed the cytocompatibility of the AgNP-modified cements using MC3T3-E1 osteoblastic cells. Cell viability and proliferation assays showed that the cements containing AgNPs exhibited no significant cytotoxicity in either the short- or long-term [2].

Despite these advances, there remains a need for multifunctional composite systems that combine biodegradable polymeric matrices with tunable inorganic phases and controlled incorporation of silver nanostructures. In particular, comparative investigations of the influence of different biocompatible stabilizing agents on AgNP performance in calcium phosphate-based composites remain limited. Thus, the present work aimed to develop antibacterial calcium polyphosphate/alginate (CPP/Alg) composites incorporating AgNPs stabilized by either alginate or polyphosphate. AgNPs were synthesized via aqueous chemical reduction using biocompatible molecules (Alg and polyphosphate) as stabilizing agents, enabling a comparative analysis of polymer- and polyphosphate-mediated systems. The CA-AgNP composites were obtained by alginate coacervate formation followed by CPP precipitation in water. The nanoparticles and composites were characterized for their physicochemical and morphological properties, and their antibacterial activity was systematically evaluated in vitro against *S. aureus* and *S. epidermidis*.

## 2. Results and Discussion

### 2.1. Preparation and Characterization of AgNPs

**Figure 1:**
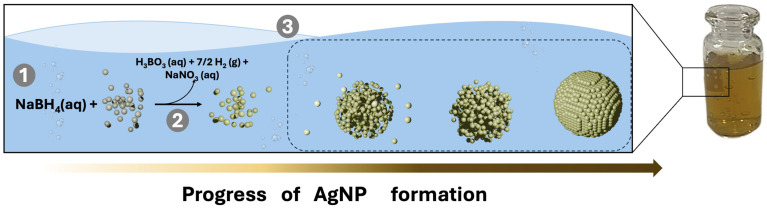
Schematic representation of the AgNP synthesis process. Step 1: reduction of Ag^+^ ions by NaBH_4_. Step 2: formation of metallic silver atoms. Step 3: nucleation and early growth of silver nanoparticles.

AgNPs were synthesized via NaBH_4_-mediated chemical reduction of silver ions (Ag^+^) [11], employing two distinct stabilizing agents: alginate (Alg) and polyphosphate (PP). The formation mechanism of AgNPs is schematized in Figure 1, which shows the sequential processes involved in obtaining the nanoparticles. The process initiates with the NaBH_4_-mediated reduction of Ag^+^ ions, leading to the formation of metallic silver atoms (Steps 1 and 2). As the concentration of these atoms increases, they collide and cluster, forming nucleation centers that define the early structure of AgNPs (Step 3). This nucleation event governs the subsequent growth and physicochemical characteristics of the nanoparticles [12,13,14]. The Ag^+^ ions reduction was also evidenced by a color change in the system to yellow.

**Figure 2:**
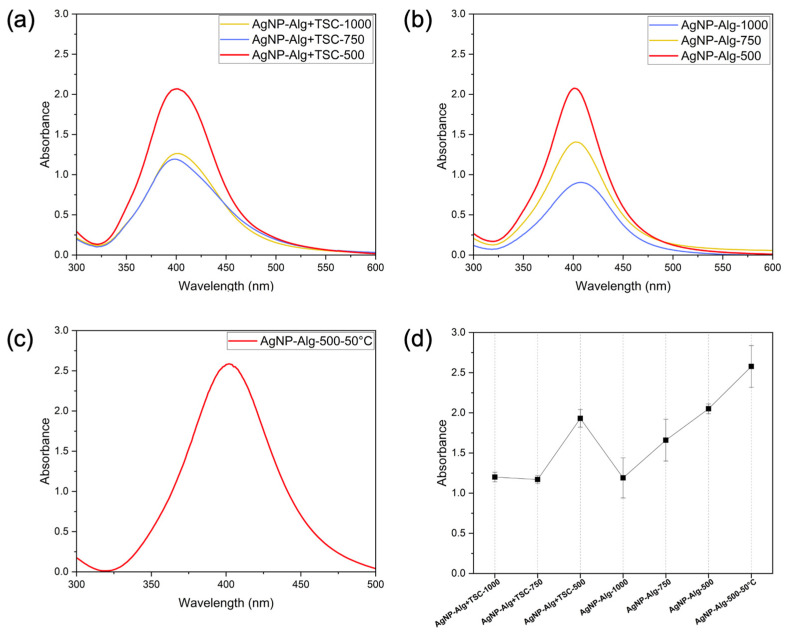
UV-Vis extinction spectra in the study of the influence of (**a**) Alg concentration with TSC, (**b**) Alg concentration, and (**c**) temperature on the synthesis of Alg-coated AgNPs. (**d**) Comparison curve of the maximum intensities of all synthesized AgNP-Alg samples.

AgNPs’ formation was confirmed by UV-Vis spectroscopy [15]. Figure 2 illustrates the UV-Vis spectra of synthesized AgNP-Alg, showing extinction bands centered at approximately 400 nm, which correspond to the localized surface plasmon resonance (LSPR) characteristic of spherical silver nanoparticles [16].

The influence of synthesis parameters on the optical response of AgNP-Alg systems was systematically evaluated, considering (i) the presence of TSC, (ii) Alg concentration, and (iii) reaction temperature. Figure 2a presents the UV-Vis extinction spectra of AgNP-Alg synthesized with different Alg volumes (1000, 750, and 500 µL) in the presence of TSC, demonstrating that variations in Alg concentration significantly affected the plasmonic band. The colloidal suspensions AgNP-Alg + TSC-1000 and AgNP-Alg + TSC-750 exhibited lower intensity compared to the AgNP-Alg + TSC-500 sample.

In another assay, we also evaluated the influence of Alg concentration (AgNP-Alg-1000, AgNP-Alg-750, and AgNP-Alg-500 samples) on the formation and stability of AgNP-Alg, without adding TSC (Figure 2b). The results showed that alginate concentrations affect the intensity of the plasmonic band. The AgNP-Alg-1000 suspension exhibited the lowest extinction intensity, followed by AgNP-Alg-750, while AgNP-Alg-500 showed the highest intensity. This trend confirms that the intensity decreases as the Alg concentration increases. Additionally, TSC did not appear to affect AgNP synthesis. The similarity between AgNP-Alg + TSC-500 and AgNP-Alg-500 suggests that TSC does not play a decisive role in nanoparticle formation within this system (Figure 2). This observation is consistent with previous reports indicating that, under strong reducing conditions, the influence of citrate is less pronounced than that of NaBH_4_ and Ag^+^ [17].

The effect of temperature on nanoparticle formation was further investigated by conducting the synthesis at 50 °C under the same conditions used for AgNP-Alg-500. The resulting UV-Vis spectrum showed an enhancement in the plasmon band intensity compared with that obtained at room temperature (Figure 2c). This change in the optical response may be associated with differences in nanoparticle concentration, particle size, and/or dispersion state [18,19]. Since the maximum wavelength and overall spectral profile remained essentially unchanged, the data suggest that increasing the reaction temperature favored nanoparticle formation without substantially altering their optical properties, although UV-Vis data alone do not allow direct quantification of synthetic yield [20].

**Figure 3:**
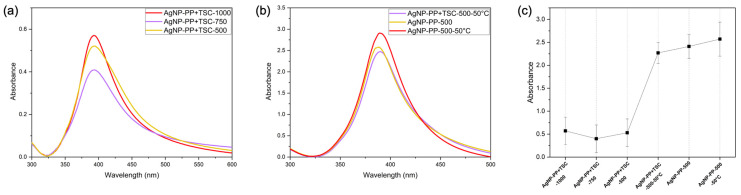
UV-Vis extinction spectra in the study of the influence of (**a**) PP concentration with TSC, (**b**) temperature on the synthesis of PP-coated AgNPs. (**c**) Comparison curve of the maximum intensities of all synthesized AgNP-PP samples.

In another approach, AgNPs stabilized with polyphosphate (PP) were synthesized under six different experimental conditions (Figure 3). Initially, TSC 75 mmol L^−1^ was employed at a constant volume (500 μL), while the PP concentration was varied (1000, 750, and 500 μL). As evidenced by the plasmon band intensities (Figure 3a), the samples AgNP-PP + TSC–1000, AgNP-PP + TSC-750, and AgNP-PP + TSC-500 exhibited only minor variations. These results indicate that variations in PP concentration do not significantly affect the plasmon band intensity, suggesting a limited influence of PP content on the optical response of the AgNPs under these synthesis conditions. Consequently, the AgNP-PP + TSC-500 system was selected for subsequent studies because it provided comparable optical performance while using a lower amount of reagents.

When the reaction temperature was increased to 50 °C (AgNP-PP + TSC-500-50 °C), a significant increase in LSPR intensity was observed, indicating that temperature plays a significant role in AgNP formation and growth (Figure 3). Furthermore, when TSC was removed from the synthesis (AgNP-PP-500), a slight increase in intensity was measured, suggesting that citrate is not essential for nanoparticle formation under the strong reducing conditions employed. Notably, the combined effect of TSC removal and heating (AgNP-PP-500-50 °C) resulted in the highest LSPR intensity, highlighting temperature as the dominant factor influencing nanoparticle formation and the plasmonic response of AgNP–PP systems.

Based on the UV-Vis spectra, the colloidal suspensions AgNP-Alg-500, AgNP-Alg-500-50 °C, and AgNP-PP-500-50 °C were selected for subsequent characterizations, as they exhibited well-defined plasmon bands centered at 400 nm and higher optical intensities than the other systems. In contrast, samples synthesized in the presence of TSC were not further investigated, as no significant improvement in plasmon band intensities was observed compared with TSC-free systems. Therefore, citrate was excluded from subsequent studies to simplify the formulation without compromising nanoparticle formation.

**Figure 4:**
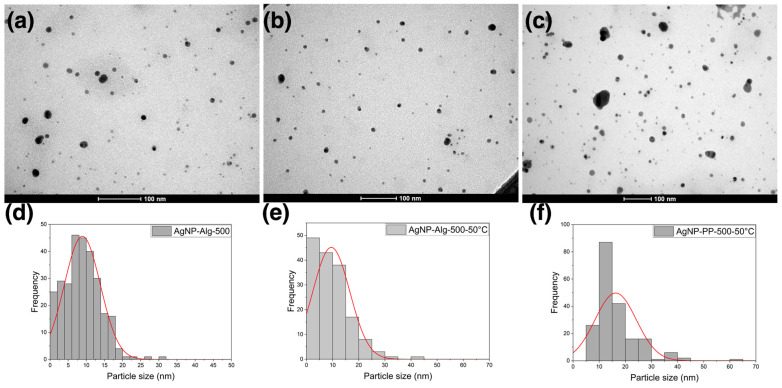
TEM images of (**a**) AgNP-Alg-500, (**b**) AgNP-Alg-500-50 °C, and (**c**) AgNP-PP-500-50 °C showing spherical AgNPs at 100 nm scale. Histogram of size distribution of (**d**) AgNP-Alg-500-50 °C, (**e**) AgNP-Alg-500, and (**f**) AgNP-PP-500-50 °C.

The UV–Vis spectra obtained for the AgNP–Alg and AgNP–PP systems exhibited a plasmon band centered at approximately 400 nm, which is characteristic of the LSPR of spherical AgNPs [15,21]. However, although UV-Vis spectroscopy provides indirect evidence of nanoparticle shape and dispersion, it does not allow direct visualization of morphology or size distribution. Therefore, TEM was employed to confirm the nanoparticle morphology, determine particle size, and assess the homogeneity of the synthesized AgNP systems [22,23]. Figure 4 shows the TEM micrographs of AgNP-Alg-500-50 °C, AgNP-Alg-500, and AgNP-PP-50 °C. The micrographs show that AgNPs are spherical, consistent with the UV-Vis data. Furthermore, the colloidal suspensions presented sizes of 9 ± 5 nm (AgNP-Alg-500-50 °C), 9 ± 7 nm (AgNP-Alg-500) and 16 ± 8 nm (AgNP-PP-50 °C) (Figure 4). These results demonstrated that the methodology is effective for synthesizing spherical AgNPs.

The measured Zeta potential showed that the AgNPs exhibited negative values, ranging from −39 to −23 mV (Table 1). Zeta potential values greater than 20 mV indicate moderately high colloidal stability due to electrostatic repulsion and the high dispersibility of the NPs, which prevent agglomeration and flocculation. Thus, the results obtained indicate that all the synthesized AgNPs exhibit colloidal stability. Furthermore, the negative surface charge is consistent with the presence of carboxylate (Alg) or phosphate (PP) groups at the nanoparticle interface, as reported in previous studies of the synthesis of AgNPs coated with Alg and PP [24,25,26].

Hydrodynamic diameters (dH) determined by DLS were significantly larger than the TEM-derived core sizes (Table 1), which is expected due to the contribution of the hydrated polymeric shell and possible nanoparticle clustering in aqueous suspension. Among the systems, AgNP-Alg-500-50 °C exhibited the largest dH (143 ± 5 nm) and a PdI of 0.77, suggesting the presence of aggregates or a broader size distribution. In contrast, AgNP-PP-500-50 °C showed the smallest dH (25.2 ± 0.2 nm), consistent with more compact stabilization.

In parallel with the ζ and dH data, Table 1 details the polydispersity index (PdI) of the AgNPs. The PdI is a dimensionless parameter used to describe non-uniform systems in terms of particle size, with values above 0.7 indicating a wide particle size distribution, while values below 0.05 indicate a highly monodisperse system [27]. The AgNP-Alg-500 and AgNP-PP-500-50 °C systems have <0.7, indicating dispersion acceptable for colloid suspensions. However, AgNP-Alg-500-50 °C shows a PdI of 0.770 ± 0.03, slightly above 0.7, indicating moderate to high polydispersity.

Although the DLS-derived PdI values were higher than those typically expected for highly monodisperse systems, similar values have been reported for polymer-stabilized AgNP or complex colloidal systems, where DLS captures the hydrodynamic size distribution and may be influenced by the solvation layer and minor aggregation. In such cases, TEM and zeta potential can provide additional information to confirm nanoscale particle formation and colloidal stability [28,29,30]. This behavior is consistent with polymer-stabilized nanoparticle suspensions, in which the hydrodynamic diameter includes the solvation layer and may be influenced by weak aggregation or the polymer size heterogeneity [27,28]. Therefore, the PdI should be interpreted together with complementary characterization techniques. In the present study, TEM analysis confirmed the formation of nanosized spherical AgNPs, while the zeta potential results indicated good colloidal stability, supporting the successful synthesis of stable nanoparticle suspensions.

### 2.2. Characterization of PP-Alg-AgNP Composites

The composite materials were prepared with two concentrations of Alg solution, 0.011 g mL^−1^ and 0.017 g mL^−1^, resulting in systems labeled C1 and C2, respectively. The colloidal systems AgNP-Alg-500, AgNP-Alg-500-50 °C, and AgNP-PP-500-50 °C were used to prepare six composite materials: C1-AgNP-Alg-500, C1-AgNP-Alg-500-50 °C, C1-AgNP-PP-500-50 °C, C2-AgNP-Alg-500, C2-AgNP-Alg-500-50 °C and C2-AgNP-PP-500-50 °C.

**Figure 5:**
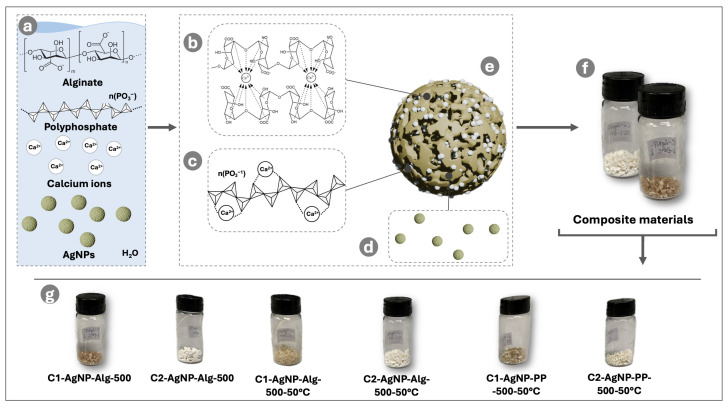
Schematic illustration of the composite material preparation using (**a**) Alg, PP, and Ca^2+^ and AgNPs. The process involves (**b**) calcium complexation by alginate, followed by (**c**) the reaction between calcium ions and PP, resulting in the formation of a (**d**,**e**) solid composite (rendered representation) with AgNPs. The final composite material is shown in (**f**,**g**).

As illustrated in Figure 5, the composite preparation involved the use of Alg, PP, and Ca^2+^ ions. The formation of the composites was governed by calcium–alginate coordination (egg-box model) and Ca^2+^–PP precipitation, yielding solid, quasi-spherical materials. Visual inspection revealed slight color variations depending on the alginate content and the incorporated AgNP system.

**Figure 6:**
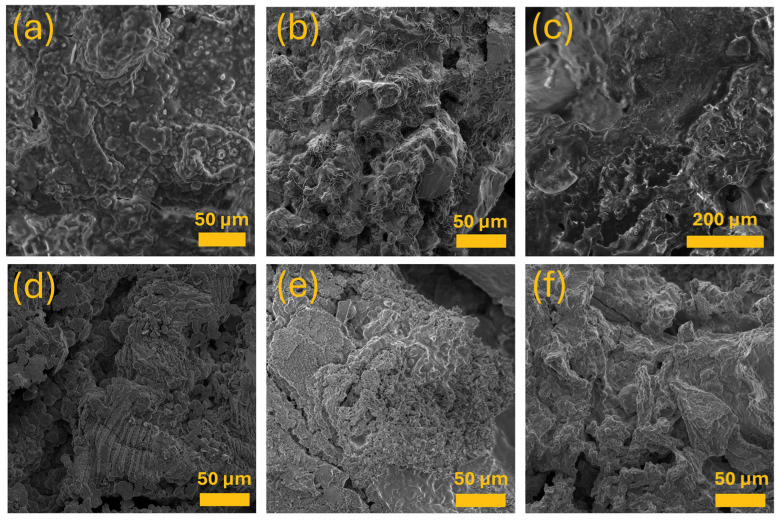
Scanning electron microscopy images of material samples (**a**) C1-AgNP-Alg-500, (**b**) C2-AgNP-Alg-500. (**c**) C1-AgNP-Alg-500-50 °C, (**d**) C2-AgNP-Alg-500-50 °C, (**e**) C1-AgNP-PP-500-50 °C, and (**f**) C2-AgNP-PP-500-50 °C showing rough surfaces with some clusters in different scales.

SEM analysis (Figure 6) revealed dense and rough surface morphologies for all synthesized composites, with no marked morphological differences observed between C1 and C2 formulations. These results suggest that alginate concentration did not strongly alter the macroscopic surface topography under the evaluated conditions.

**Figure 7:**
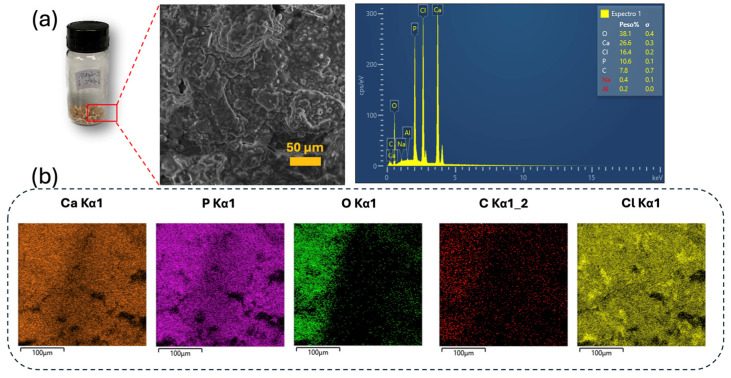
(**a**) SEM image and EDS spectra and (**b**) elemental maps of a composite material (C1-AgNP-Alg-500). EDS shows the composition of the areas in the SEM image.

To analyze the chemical composition of the composites, the elemental maps and EDS spectra of the surface were obtained (Figure 7). The EDS elemental analysis revealed a high content of calcium (Ca), phosphorus (P), oxygen (O), carbon (C), and chlorine (Cl) in the designated region of the SEM image. The identification of high levels of Ca, P, O, and C confirms the formation of the Alg- and PP-based composite. Additionally, the presence of chlorine was attributed to chloride ions (Cl^−^) from CaCl_2_ used during ionic crosslinking. The EDS results presented in Figure 7 are representative of all experimental materials.

However, elemental mapping did not detect Ag signals, which may be attributed to the low Ag content and EDS’s limited sensitivity for trace-level detection. Therefore, ICP-OES was used to quantify Ag content in the composites. The materials exhibited varying Ag concentration values (Table 2). The Ag content was then correlated with the composite mass used for digestion, and it was observed that all composites showed a comparable Ag mass fraction of 0.002–0.003% (Table 2). Therefore, ICP-OES analysis confirmed the presence of Ag in the composites.

Although the measured Ag content in the composites was relatively low, direct comparison with previously reported Ag-containing biomaterial remains challenging because many studies do not quantify or report the actual silver content incorporated into the final composite [7]. In the present work, ICP-OES was used to quantify the Ag mass fraction, which enabled a more direct assessment of the relationship between silver loading and antibacterial response. Notably, the composites retained measurable antibacterial activity despite the low Ag content, supporting the functional relevance of the incorporated silver phase.

**Figure 8:**
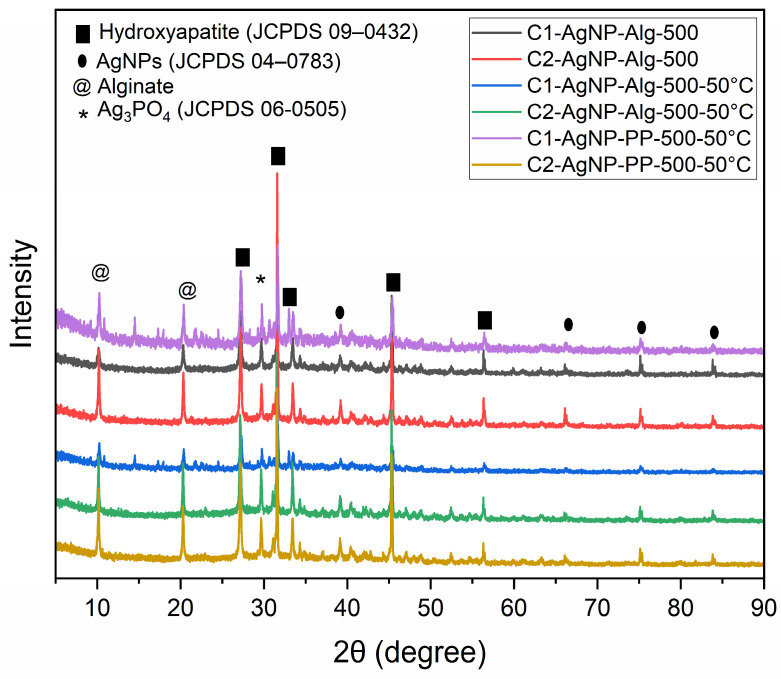
X-ray diffraction patterns of the composites in the 5–90° 2θ range.

To investigate the crystalline phases present in the composites, X-ray diffraction (XRD) analysis was performed. Figure 8 shows the diffraction patterns of the composites. The XRD patterns predominantly exhibited peaks at 27.2°, 31.6°, 33.5°, 45.3°, and 56.3°, corresponding to (0 0 2), (2 1 1), (3 0 0), (2 2 2), and (0 0 4) planes of the crystalline phases of hydroxyapatite (JCPDS no.09–0432) [31,32]. The XRD pattern also presented the peaks at 39.2°, 66.2°, 75.2°, and 83.5°, with lower intensity, which can be attributed to the (1 1 1), (2 2 0), (3 1 1), and (2 2 2) planes of the face-centered cubic structure of metallic silver, indicating the incorporation of AgNPs [33,34]. In addition, the peaks presented at 10.2° and 20.4° are from the semi-crystalline structure of alginate composites [35]. The presence of a peak at 29.7°, which does not match any of the previous structures, and is related to the formation of Ag_3_PO_4_ structures (JCPDS 06-0505) [36,37], suggests that Ag^+^ ions partially substituted Ca^2+^ in phosphate clusters. However, a stronger presence of crystalline HAp phases was still observed, indicating that interactions between silver and phosphate ions occurred on a smaller scale.

**Figure 9:**
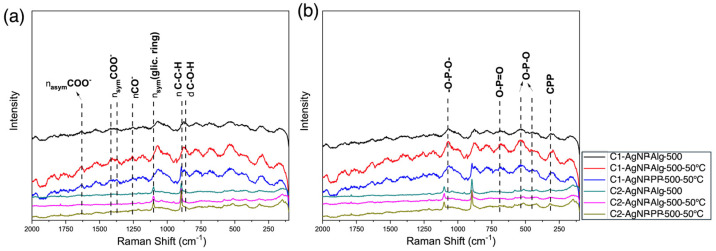
Raman spectra with assigned vibrational bands corresponding to (**a**) Ag–Alg interactions and (**b**) polyphosphate (PP) species.

To investigate vibrational signals related to material composition, Raman spectroscopy was performed (Figure 9). The spectra were recorded over 100–4000 cm^−1^, with band assignments concentrated in the 100–2000 cm^−1^ window. The Raman spectra of alginate can generally be divided into two regions: below 1300 cm^−1^ and above 1300 cm^−1^. The first region is mainly attributed to vibrations of the polymer backbone, whereas the latter is commonly associated with stretching modes of carboxylic groups [38,39]. In addition, band shifts related to the interaction between Ag and Alg were expected, particularly in vibrational modes associated with β-D-mannuronic acid residues, since α-L-guluronic acid residues are more prone to interact with Ca^2+^ [39]. Based on the results, the presence of AgNPs was highlighted through band assignments corresponding to interactions between Ag and the matrix at 870, 877, 1103, 1257, 1372, 1400, and 1630 cm^−1^ [38,40].

Regarding the inorganic matrix, a set of bands was attributed to CPP, as shown in Figure 9b. Specifically, Ca^2+^ coordinated within a polyphosphate (PP) arrangement was identified at 332 cm^−1^. Low Raman shift bands were assigned to O–P–O vibrational modes, observed at 477 and 530 cm^−1^ [41]. The symmetric stretching peaks (O–P=O) and (–O–P–O–) at 689 and 1179 cm^−1^, respectively, were attributed to linear and terminal phosphate groups [42].

### 2.3. Antibacterial Assays

**Figure 10:**
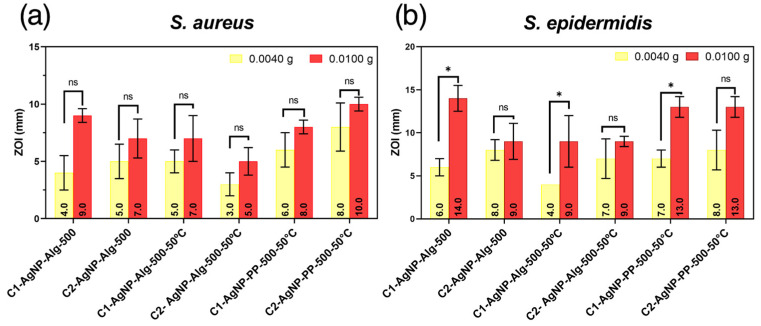
Comparative plots of the ZOI (mm) as a function of the mass (0.0040 g and 0.010 g) against (**a**) *S. aureus* and (**b**) *S. epidermidis*. Error bars present mean ± standard deviation (n = 3). ns = statistically non-significant and * statistically significant (*p* < 0.05).

**Figure 11:**
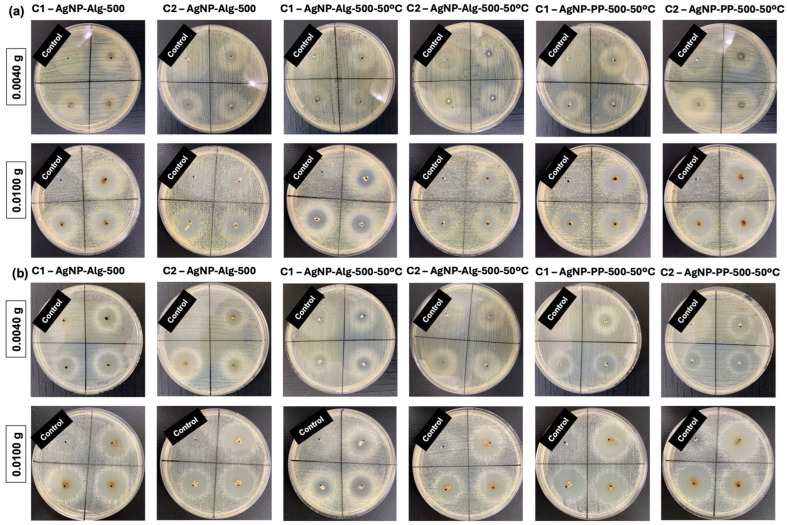
Diameter of ZOI (mm) obtained by agar diffusion assay against (**a**) *S. aureus* and (**b**) *S. epidermidis*, showing the composites tested at two composite masses: 0.004 and 0.01 g.

Antibacterial activity evaluation is essential to assess the effectiveness of Ag-containing biomaterials intended for bone regeneration, where infection control is critical [43,44]. Therefore, antibacterial tests were performed to assess the inhibitory potential of the developed composites against *S. aureus* and *S. epidermidis*. Overall, the findings demonstrate that all composite materials exhibit antibacterial potential. The results are summarized in Figure 10 and Table S1, and the corresponding photographs of the samples incubated with the bacterial strains are presented in Figure 11.

A qualitative comparison of the zones of inhibition (ZOI) revealed differences among the tested materials (Figure 11). Notably, the C2-AgNP-PP-500–50 °C composite exhibited the largest inhibition halos, indicating enhanced antibacterial performance. In contrast, composites containing AgNP-Alg-500-50 °C produced comparatively smaller inhibition halos. When these results are considered together with the Ag contents in the composites (Table 2), it is clear that antibacterial activity was observed even at relatively low silver loadings. Interestingly, the composites containing AgNP-PP-500–50 °C showed higher antibacterial activity, despite the fact that they did not contain the highest Ag content. This finding suggests that antibacterial performance is not determined solely by the total amount of silver incorporated into the composites. Rather, it may also depend on the stabilizing matrix and on differences in silver accessibility and release behavior, which can influence interactions between the composite and bacterial cells [45,46]. Nevertheless, because the present study focused on preliminary antibacterial screening, the main mechanism(s) underlying this activity cannot yet be established and will require dedicated silver-release kinetics and mechanistic studies in future work.

In this study, the control group consisted of a composite based on alginate and calcium polyphosphate (Alg/CPP) without AgNPs, to evaluate the direct contribution of AgNPs to the antibacterial activity of the developed materials. The control composite did not exhibit inhibition zones against either *S. aureus* or *S. epidermidis*, confirming that the antibacterial effect observed in the composites is associated with the presence of AgNPs.

To further assess the influence of sample mass on antibacterial activity, a one-way ANOVA (95% confidence level) was performed. The results indicated statistically significant differences only for the assays conducted against *S. epidermidis*, specifically for the composites C1–AgNP–Alg–500, C1–AgNP–Alg–500–50 °C, and C1–AgNP–PP–500–50 °C (Figure 10). This finding suggests that, for this bacterial strain, the tested mass influences the antibacterial effectiveness depending on the composite formulation, whereas no significant effect was observed for *S. aureus*. Such behavior may be associated with differences in cell wall structure and susceptibility between the bacterial species, with *S. epidermidis* potentially being more sensitive to variations in silver ion availability.

The study of Ag content is an important procedure for evaluating the antibacterial potential of CPP- and AgNP-based composites. Andrade et al., for example, investigated the antibacterial properties of HAp/AgNP composites and reported that a Ag content higher than 0.024 ± 0.002% relative to the sample mass is required to inhibit the activity of *S. aureus* (ATCC 25923) and *E. coli* (ATCC 25922). However, compared with the results reported by Andrade et al., the CPP-Alg-AgNP composites produced in this study have lower Ag contents and still exhibit satisfactory antibacterial activity [47].

In the present study, our primary aim was to develop and characterize antibacterial biocomposites and to conduct an initial proof-of-concept assessment of their antimicrobial activity. Although biocompatibility, cytotoxicity, and osteogenic performance are essential parameters for future translational studies, these aspects were beyond the scope of the current work and will be investigated in subsequent studies aimed at establishing the biological safety and bone regenerative potential of the developed composites.

A limitation of the present study is that the antibacterial activity of the composites was evaluated only by agar-diffusion assays, which provide an initial assessment of antimicrobial activity but do not fully reflect the complexity of clinical bone infections. Since biofilm formation plays a critical role in postoperative and implant-associated infections, future studies should include dedicated anti-biofilm assays to evaluate both the prevention of biofilm formation and the disruption of established biofilms [48,49]. Such investigations will provide a more comprehensive understanding of the potential of these composites for orthopedic and bone tissue engineering applications.

In summary, these results are promising for the continued development of antibacterial composite-based materials for biomedical applications, particularly in contexts where infection prevention is crucial. Nevertheless, future in vivo studies will be essential to further evaluate biocompatibility, degradation behavior, and bone-regeneration potential under physiologically relevant conditions.

## 3. Materials and Methods

### 3.1. Chemical Reagents

Sodium alginate (Alg, [C_6_H_7_(NaO)_6_]_n_, 90%, Dinâmica Química Contemporânea LTDA, Indaiatuba, Brazil), sodium borohydride (NaBH_4_, ≥98.0%, Sigma-Aldrich, São Paulo, Brazil), trisodium citrate (TSC, Na_3_C_6_H_5_O_7_·H_2_O, 90%, Dinâmica Química Contemporânea LTDA, Indaiatuba, Brazil), calcium chloride (CaCl_2_, ≥96%, Sigma-Aldrich, São Paulo, Brazil), sodium hexametaphosphate (PP, 96%, Sigma-Aldrich, São Paulo, Brazil), silver nitrate (AgNO_3_, ≥99.0%, Sigma-Aldrich, São Paulo, Brazil) and ultrapure water (18.2 MΩ·cm of resistivity, room temperature).

### 3.2. Synthesis of Alginate-Coated Silver Nanoparticles

The synthesis of AgNPs stabilized with alginate (AgNP-Alg) was carried out using the chemical reduction method, varying the experimental conditions and using different volumes of Alg (500, 750, and 1000 µL) to improve the reaction yield.

Initially, 50 µL of AgNO_3_ solution (50 mmol L^−1^) was added to 24 mL of ultrapure water. Then, an adequate volume of TSC (75 mmol L^−1^) or sodium alginate (5 mg mL^−1^) was added according to the conditions described in Table 3. After complete homogenization of the reagents, 250 µL NaBH_4_ (100 mmol L^−1^) was added to the reaction. The mixture was stirred for approximately 5 min at room temperature (~20–25 °C) or 50 °C (Table 3).

The AgNP-Alg samples were purified by centrifugation at 1110× *g* for 5 min using 10 kDa MWCO ultrafiltration tubes (Vivaspin^®^, GE Healthcare, Chicago, IL, USA) in a centrifuge (Cence TD3, Xiangyi, Changsha, China), thereby simultaneously purifying and concentrating the colloidal suspensions. The initial AgNP dispersions (25 mL) were concentrated to approximately 10 mL. These purified and concentrated suspensions were subsequently employed for physicochemical characterization and composite preparation.

### 3.3. Synthesis of Polyphosphate-Coated Silver Nanoparticles

The AgNPs stabilized with PP were also prepared, varying the synthesis parameters (Table 4). To achieve AgNP-PP, 50 µL of AgNO_3_ (50 mmol L^−1^), 500 µL of TSC (75 mmol L^−1^), and PP (5 mg mL^−1^) were added to 24 mL of ultrapure water. After complete homogenization, 250 µL of NaBH_4_ (100 mmol L^−1^) was added to the reaction medium. The mixture was stirred for approximately 5 min at RT (20–25 °C) or 50 °C.

The AgNP-PP samples were subjected to the same centrifugation and ultrafiltration protocol described for AgNP-Alg, using 10 kDa MWCO ultrafiltration tubes (Vivaspin^®^, GE Healthcare, Chicago, IL, USA). These purified and concentrated suspensions were subsequently employed for physicochemical characterization and composite preparation.

### 3.4. Preparation of PP-Alg-AgNPs Composites

The composite material was obtained through the interaction between calcium ions (Ca^2+^) and alginate, as well as the precipitation reaction between the phosphate groups of PP and Ca^2+^, followed by the addition of the colloidal suspensions AgNP-Alg-500, AgNP-Alg-500-50 °C, or AgNP-PP-500-50 °C.

The synthesis was conducted via coacervate formation. Initially, two sodium alginate solutions were prepared: (i) 0.011 g mL^−1^ and (ii) 0.017 g mL^−1^, dissolving the Alg at 50 °C under continuous stirring. Approximately 0.5 g of PP, previously dissolved in 5 mL of ultrapure water, was added to each Alg solution. The materials obtained from Alg solutions (i) and (ii) were designated as C1 and C2, respectively. The system was homogenized for 5 min to ensure component interaction.

Subsequently, 1 mL of the purified AgNP suspension (AgNP-Alg-500, AgNP-Alg-500-50 °C, and AgNP-PP-500-50 °C) was added to the Alg-PP mixture. The system was maintained under homogenization for 5 min at RT. The mixture was dripped into 10 mL of CaCl_2_ (1.0 mol L^−1^). As the droplets entered the CaCl_2_ solution, spherical solid structures were formed. The resulting solids were separated from the Ca^2+^ solution and transferred to an oven, where they were dried at 100 °C for approximately 2 h.

### 3.5. Materials Characterization

#### 3.5.1. Ultraviolet–Visible Absorption Spectroscopy

To perform UV-Vis absorption spectroscopy, 1 mL of AgNP suspension was transferred to a quartz cuvette, and the volume was completed to 3 mL with ultrapure water. The absorption profiles were acquired using an Evolution 600 s spectrophotometer (Thermo Scientific, Waltham, MA, USA) with a scan range of 300–600 nm and a resolution of 1 nm.

#### 3.5.2. Transmission Electron Microscopy

The morphology and size distribution of the nanoparticles were investigated using transmission electron microscopy (TEM). The micrographs were obtained using the transmission electron microscope FEI Tecnai G2 Spirit Biotwin (Thermo Fisher Scientific, Hillsboro, OR, USA) with a resolution of 0.34 nm, operated at 120 kV. The obtained data were analyzed by ImageJ version 1.53k (National Institutes of Health, USA), using at least 100 nanoparticles to estimate the AgNP size.

#### 3.5.3. Zeta Potential and Dynamic Light Scattering

The AgNPs’ hydrodynamic diameter and the Zeta potential (ζ) were determined using a Zetasizer Nano ZS90 (Malvern Panalytical, Worcestershire, UK). Approximately 100 μL of purified AgNP suspensions were dispersed in 1 mL of ultrapure water and subsequently analyzed by Zeta potential and dynamic light scattering (DLS) measurements.

#### 3.5.4. X-Ray Diffraction

X-ray diffraction (XRD) analysis of the composite materials was performed using an XRD-7000 diffractometer (Shimadzu, Kyoto, Japan) with CuKα radiation (1.5418 Å) and an operating voltage of 40 kV. The diffraction patterns were recorded over a 2θ range from 5° to 90°. For this analysis, the materials were ground using an agate mortar and pestle, and kept at room temperature.

#### 3.5.5. Scanning Electron Microscopy

Scanning electron microscopy (SEM, Mira3 scanning electron microscope from Tescan, Brno, Czech Republic) was used to investigate the surface morphology of the composites. The samples were set on carbon tape and coated with gold.

#### 3.5.6. Attenuated Total Reflectance Fourier-Transform Infrared Spectroscopy

Attenuated total reflectance Fourier-transform infrared (ATR-FTIR) spectra were used to identify functional groups in the composite material samples. The spectra were obtained on a PerkinElmer FTIR spectrum 3 with UATR accessory (Shelton, CT, USA).

#### 3.5.7. Raman Scattering Spectroscopy

Raman spectra were acquired using a Raman-AFM spectrometer, model iHR320 (Horiba, Kyoto, Japan), equipped with an integrated microscope. The instrument was operated with a 671 nm laser at 40 mW. Spectra were collected in the 100–2000 cm^−1^ range with a scan speed of 159 nm s^−1^ and step size of 1.0 nm.

#### 3.5.8. ICP-OES

Inductively coupled plasma optical emission spectroscopy (ICP-OES, iCAP 6300, Thermo Scientific^,^ Waltham, MA, USA) was used to determine the silver content in the composites. A determined mass of the composite materials was mixed with 0.38 mL of 65% HNO_3_ followed by the addition of ultrapure water, ensuring a final acid concentration of 5% in water in a total volume of 5 mL. Subsequently, 1 mL of H_2_O_2_ was added to the system, which was then subjected to ultrasound for 30 min. After this period, the system was diluted to a final volume of 10 mL with ultrapure water and again subjected to ultrasound for 15 min. The ICP-OES analysis was performed using a calibration curve prepared with a silver standard solution (detection wavelength: 328.068 nm).

### 3.6. Antibacterial Assays

The composite’s antibacterial activity was assessed by inhibition zone tests against *S. aureus* and *S. epidermidis*. The bacterial strains were inoculated into 5 mL of tryptic soy broth (TSB) and incubated at 37 °C for 24 h. After that, the bacterial cultures were centrifuged at 2495× *g* for 5 min (Universal Centrifuge 320 R, Hettich Zentrifugen, Andreas Hettich GmbH, Tuttlingen, Germany). The supernatant was discarded under laminar flow conditions, and the pellet was resuspended in 5 mL of sterile saline solution. The optical density (OD) of the bacterial suspension was adjusted to 1, corresponding to 3 × 10^8^ CFU/mL on the McFarland scale [50,51]. The outer surface of a Petri dish was divided into four sections, one designated as the control (composite based on Alg + CPP) and the remaining three for the AgNP samples, with triplicate results for each AgNP sample type. The bacterial suspension with adjusted OD was spread in a lawn pattern on the surface of a Petri dish containing approximately 25 mL of nutrient agar, using a sterile swab under laminar flow conditions. The AgNP samples were applied with sterile forceps, preferably flame-sterilized or sterilized with 70% alcohol. After sealing the Petri dish, it was incubated in a microbiological incubator at 37 °C for 24 h. Finally, the presence of an inhibition zone around the applied samples was evaluated by measuring its diameter with a ruler. The Petri dish was then sealed with Parafilm, wrapped with aluminum foil or plastic film, and stored at 4 °C in a refrigerator for further analysis. The assays were performed in triplicate.

### 3.7. Statistical Analysis

GraphPad Prism (version 8, GraphPad Software, San Diego, CA, USA) was used for statistical analyses. The obtained data were expressed as mean ± standard deviation (SD). One-way analysis of variance (ANOVA) followed by Tukey’s multiple comparison test was used to assess statistically significant differences among the experimental groups. Differences were considered statistically significant when *p* < 0.05.

## 4. Conclusions

Silver nanoparticles were successfully synthesized via NaBH_4_-mediated reduction and stabilized using alginate or polyphosphate matrices. UV–Vis spectroscopy indicated the formation of localized surface plasmon resonance bands characteristic of quasi-spherical AgNPs, which was corroborated by TEM analysis confirming nanoscale spherical morphology. The nanoparticles exhibited negative Zeta potential values and hydrodynamic diameters consistent with colloidally stable dispersions. CPP-Alg-AgNP composites were subsequently prepared through ionic crosslinking and coacervation. XRD analysis revealed the predominance of the crystalline hydroxyapatite phase, while Raman spectra confirmed bands associated with both the inorganic phase and the polymer. SEM micrographs showed irregular but homogeneous morphology. The silver contents in the composites ranged from 0.390 ± 0.002 to 0.2180 ± 0.0002 mg L^−1^.

The agar diffusion assays confirmed the antibacterial activity of the composites against *S. aureus* and *S. epidermidis*, demonstrating a positive correlation between the sample mass and the inhibition halo diameter. These findings reinforce the functional role of AgNPs incorporated into CPP/Alg matrices and indicate that the composites are promising antibacterial materials. In perspective, this study paves the way for improving the physicochemical and biological properties of the composites, aiming to develop novel biomedical devices with theranostic potential.

## Figures and Tables

**Table 1:** Physicochemical properties of the AgNP-Alg-500-50 °C, AgNP-Alg-500, and AgNP-PP-500-50 °C: zeta potential, hydrodynamic radius, and polydispersity index (PdI).

Sample	ζ (mV)	d_H_ (nm)	PdI
AgNP-Alg-500-50 °C	−23.0 ± 0.3	143 ± 5	0.770 ± 0.03
AgNP-Alg-500	−38.7 ± 0.2	51.3 ± 0.6	0.531 ± 0.004
AgNP-PP-500-50 °C	−35 ± 2	25.2 ± 0.2	0.681 ± 0.008

**Table 2:** Silver contents of the composites obtained by ICP-OES.

Composite	[Ag] (mg/L) ^(a)^	m_Ag_ (µg) ^(b)^	m_composite_ (mg) ^(c)^	Ag Content (% *w*/*w*) ^(d)^
C1-AgNP-Alg-500	0.390 ± 0.002	3.90	141.5	0.003
C2-AgNP-Alg-500	0.275 ± 0.003	2.75	103.0	0.003
C1-AgNP-Alg-500-50 °C	0.2180 ± 0.0002	2.18	101.3	0.002
C2-AgNP-Alg-500-50 °C	0.35 ± 0.02	3.50	223.0	0.002
C1-AgNP-PP-500-50 °C	0.225 ± 0.003	2.25	95.7	0.002
C2-AgNP-PP-500-50 °C	0.257 ± 0.001	2.57	116.9	0.002

^(a)^ Values obtained by ICP analysis. ^(b)^ Calculated values based on the sample submitted for ICP analysis. ^(c)^ Mass of composite digested for ICP analysis. ^(d)^ Ag mass percentage in the composites.

**Table 3:** Experimental parameters used in the synthesis of AgNP-Alg.

Sample	Volume of TSC (µL)	Volume of Alg (µL)	Temperature
AgNP-Alg + TSC-1000	500	1000	RT ^1^
AgNP-Alg + TSC-750	500	750	RT
AgNP-Alg + TSC-500	500	500	RT
AgNP-Alg-1000	0	1000	RT
AgNP-Alg-750	0	750	RT
AgNP-Alg-500	0	500	RT
AgNP-Alg-500-50 °C	0	500	50 °C

^1^ RT: ~20–25 °C.

**Table 4:** Experimental parameters used in the synthesis of AgNP-PP.

Sample	Volume of PP (µL)	Volume of TSC (µL)	Time (min)	Temperature (°C)
AgNP-PP + TSC-1000	1000	500	5	RT ^1^
AgNP-PP + TSC-750	750	500	5	RT
AgNP-PP + TSC-500	500	500	5	RT
AgNP-PP + TSC-500-50 °C	500	500	5	50 °C
AgNP-PP-500	500	0	5	RT
AgNP-PP-500-50 °C	500	0	5	50 °C

^1^ RT: ~20–25 °C.

## Data Availability

The original contributions presented in this study are included in the article/Supplementary Material. Further inquiries can be directed to the corresponding authors.

## Supplementary Materials

The following supporting information can be downloaded at: https://www.mdpi.com/article/10.3390/ph19060917/s1. Table S1. Antibacterial test results, radius of the inhibition zone (mm), of the samples against *S. aureus* and *S. epidermidis*.
