# Supplementary material for: Biocomposites of Alginate, Calcium Polyphosphate, and Silver Nanostructures: Antibacterial Systems for Bone Regeneration Applications
Source: Pharmaceuticals (Basel). 2026 Jun 10;19(6):917. doi: 10.3390/ph19060917 (PMC13304926; doi:10.3390/ph19060917)
Supplement: Supplementary file 1 [file pharmaceuticals-19-00917-s001.zip › pharmaceuticals-4336740-supplementary.pdf]

Supplementary material

Biocomposites of Alginate, Calcium Polyphosphate, and Silver Nanostructures: Antibacterial Systems for Bone Regeneration Applications

Joalen Pereira do Monte [1], Rafael B. G. Pessoa [2], Adriana Fontes [3], Beate S. Santos [4], Giovannia A. L. Pereira [1,*] and Goreti Pereira [5,*]

Table S1. Antibacterial test results, radius of the inhibition zone (mm), of the samples against *S. aureus* and *S. epidermidis*.

Composite	Zone of the inhibition (mm) *S. aureus* $m_{composite}$ (g) 0.0040	*S. aureus* $m_{composite}$ (g) 0.0100	*S. epidermidis* $m_{composite}$ (g) 0.0040	*S. epidermidis* $m_{composite}$ (g) 0.0100
C1-AgNP-Alg-500	4 ± 1	9 ± 1	6 ± 1	14 ± 1
C2-AgNP-Alg-500	5 ± 1	7 ± 2	8 ± 1	9 ± 2
C1-AgNP-Alg-500-50ºC	5 ± 1	7 ± 2	4 ± 0	9 ± 3
C2- AgNP-Alg-500-50ºC	3 ± 1	5 ± 1	7 ± 2	9 ± 1
C1-AgNP-PP-500-50°C	6 ± 1	8 ± 1	7 ± 1	13 ± 1
C2-AgNP-PP-500-50°C	8 ± 2	10 ± 1	8 ± 2	13 ± 1
